# Detection of Ground Contact Times with Inertial Sensors in Elite 100-m Sprints under Competitive Field Conditions

**DOI:** 10.3390/s21217331

**Published:** 2021-11-04

**Authors:** Patrick Blauberger, Alexander Horsch, Martin Lames

**Affiliations:** 1 Chair of Performance Analysis and Sports Informatics, Technical University Munich, 80992 Munich, Germany; martin.lames@tum.de; 2 Department of Computer Science, UiT The Arctic University of Norway, 9037 Tromsø, Norway; alexander.horsch@uit.no

**Keywords:** running, sprinting, contact time, sports analytics, inertial sensors (IMU), field application

## Abstract

This study describes a method for extracting the stride parameter ground contact time (GCT) from inertial sensor signals in sprinting. Five elite athletes were equipped with inertial measurement units (IMU) on their ankles and performed 34 maximum 50 and 100-m sprints. The GCT of each step was estimated based on features of the recorded IMU signals. Additionally, a photo-electric measurement system covered a 50-m corridor of the track to generate ground truth data. This corridor was placed interchangeably at the first and the last 50-ms of the track. In total, 863 of 889 steps (97.08%) were detected correctly. On average, ground truth data were underestimated by 3.55 ms. The root mean square error of GCT was 7.97 ms. Error analyses showed that GCT at the beginning and the end of the sprint was classified with smaller errors. For single runs the visualization of step-by-step GCT was demonstrated as a new diagnostic instrument for sprint running. The results show the high potential of IMUs to provide the temporal parameter GCT for elite-level athletes.

## 1. Introduction

In recent years, the acquisition of performance parameters with sensors for application in sport science and practice has been a developing topic. The evolution towards smaller and lighter devices like inertial measurement units (IMU) allows for field usage in professional sports. With the availability of increased sample rates, even sports with fast and abrupt movements get in the scope of detailed sensor-based analysis.

In sports with high speeds, such as sprints, little direct feedback is available to the athlete. Hence, scientific assistance needs to assess objective indicators to give detailed feedback and enhance future performances. Temporal parameters like ground contact time (GCT), step duration, and step rate are common features of running analysis. These parameters were linked to enhanced performances in several studies [1,2,3], underlining their helpfulness for coaches and athletes in training and competition. Additionally, the influence of GCT on the running economy was stated [4]. In sprinting, a negative correlation between GCT and performance in a sample including french elite-level sprinters was found [5]. Especially GCT is an essential parameter in sprinting [6] but is not commonly available in training and competition settings until now [7].

Temporal parameters, in which tiny deviations can play a decisive role, and can only be captured with technological aid. Established methods like video footage, timing gates, contact mats, motion capturing, or force plates are used to determine exact temporal sprint parameters [8,9,10]. The feedback obtained by these methods can improve the quality of the running technique. However, they require notable effort or preparation and cannot be commonly applied in the field or competition settings, where the most valuable information may be collected. Non-invasive position detection methods with less overall (Global positioning system—GPS) or on-court preparation (Local positioning system—LPS) can eliminate these problems. Seidl et al. investigated the determination of temporal parameters with a LPS. For the detection of ground contact times, the most precise commonly used position detection method, LPS, does not work [11,12]. This also implies that GPS is not suitable for this detection. For reliable sprint parameter detection, photoelectric systems like Optogait or Optojump are commonly used [7,12,13,14]. However, before the measurement, these systems need to be set up on the running track carefully, thus requiring a large effort and not suited for analyses of official competitions. Moreover, only straight runs of one athlete at a time can be assessed.

To substitute these time intensive and costly systems, the integration of IMUs for diagnostics in gait [15,16,17], runs [18,19,20], or sprinting [13,21,22,23,24,25] received much attention in the last decade. Various studies introduced new or adapted sprint performance metrics based on data of IMUs. In a systematic review, Macadam et al. gathered several studies investigating one or more types of temporal parameters for sprint kinematics. They conclude, among other things, that a more distal sensor placement (e.g., foot, shank, shoe-mounted) enhances the validity and reliability of sensor measurements [26]. Also, a sampling frequency of >200 Hz improved results in the examined studies. A recent study proposed combining data from a LPS with integrated IMUs positioned near the participant’s sacrum for a more holistic view of gait parameters [27]. They stated good results for speed and stride length while not addressing ground contact time. Schmid et al. investigated the validity of IMU measurements with real-time quantification of the collected data. They report detection errors of −2.5 ± 4.8 ms for GCT and a correct step detection rate of 95.7% [13]. In a recent study, Falbriard et al. investigated temporal parameters during hurdle running. Besides a perfect hurdle clearance detection (with the help of magnetic sensors) and determination of the leading leg, they found an increase in GCT during one race [28]. Schmid and colleagues suggested a discussion regarding the GCT values, mentioning a correction procedure based on the previous study of Falbriard et al. [22,29].

From the current literature, it remains unclear whether the detection of sprint parameters with IMUs can determine the GCT of elite-level 50 and 100-m sprinters in the field. Precise GCT information could be beneficial for coaches, athletes, and science to investigate training and competition success. This study aims to validate the detection of GCTs for elite sprinters in the field with shoe-mounted IMUs.

## 2. Materials and Methods

### 2.1. Sample and Protocol

The sample consists of 1140 steps from 34 maximum 50 and 100-m sprints performed by five elite national sprinters, with three participants of the Tokyo Olympics (age: 22.6 ± 2.7 years; weight: 69.6 ± 11.5 kg; 3 male, 2 female; test year’s best official 100 m time: f: 11.65 s, f: 11.11 s, m: 10.76 s, m: 10.77 s, m: 11.27 s); 889 of these steps were simultaneously measured with the photoelectric Optogait system. The trials were performed on official sprinting tracks during three separate training sessions. Before the study, all athletes were instructed verbally and received written information about the procedure and purpose of the study. The study has been approved by the ethical committee of Technical University Munich and all subjects gave informed consent.

### 2.2. Measurement Systems

**Figure 1:**
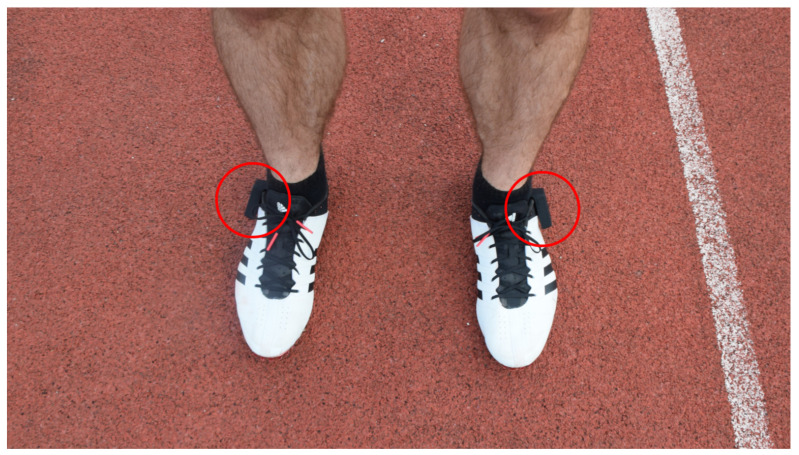
Sprint shoes with IMUs attached to the ankles (red circles).

Two IMUs (Physilog5, Gait Up SA, Lausanne, Switzerland, size: 47.5 mm × 26.5 mm × 10 mm, weight: 11 g) were attached to each athlete’s shoes, positioned right above the ankle of the foot (Figure 1). The IMUs were chosen to be easily applicable, light, and least obstructive for the athletes’ performance. The positioning was reported not to be of any problem by each athlete. The IMU included an accelerometer (512 Hz, ±16 g operating range) and gyroscope (512 Hz, ±2000°/s operating range) and a barometric sensor. In this project, only the 3D accelerometer and 3D gyroscope are used. For ground truth data acquisition, a 50 m corridor of photoelectric bars on the ground (Optogait, Bolzano, Microgate, Bolzano, Italy) was used. Optogait was used already as criterion measurement system in previous sprinting studies [7,13,30]. This system is modularly composed of 1 m bars, which can be connected to cover an arbitrary distance. Over the area of 100 cm × 80 cm, 96 light diodes are evenly located 3 mm above the running track. One validation study reported 95% limits of agreement of 7.7% for GCT with a contact mat [31], whereas another study did not find significant differences in GCT compared to a high speed video camera [32]. To acquire data for the total 100 m distance, the Optogait corridor was repositioned to the second 50 m sector for 6 of the sprints. With this procedure, ground truth data for 77.98% of the steps were captured (76.72% on the first 50 m and 23.28% steps of the last 50 m).

### 2.3. Data Processing

Initially, all raw data of both systems were exported as local text files, and personalized data was anonymized. The software MATLAB (R2021b, The MathWorks Inc., Natick, MA, USA) was used for further data processing steps. The built-in functions *butter*, *filtfilt*, and *findpeaks* were used in the algorithm. X, Y, and Z signals from the IMUs’ Accelerometer and Gyroscope were acquired at 512 Hz and extracted with the company’s software tool. The sensor’s respective information about the direction of acceleration and angular velocity is described relative to the sensor’s position, which is constantly changing during a movement. The current study summarized Accelerometer and Gyroscope outputs to one vector as vector magnitude unit (VMU) to circumvent this problem. Both VMUs were filtered using a 2nd order Butterworth low pass filter with a cut-off frequency at 70 Hz [28]. These filter parameters were successfully applied to the training data and achieved the best results. The automatic detection of step events was achieved based on two relevant episodes of a step cycle: Initial contact (IC) and terminal contact (TC). IC describes the moment in time when the heel initiates the very first contact with the ground. TC, also known as toe-off, refers to the moment when the tip of the foot leaves the ground. The precise temporal location of these events can be determined with different procedures. It turned out to be most promising to use patterns in accelerometer as well as gyroscope data [22].

**Figure 2:**
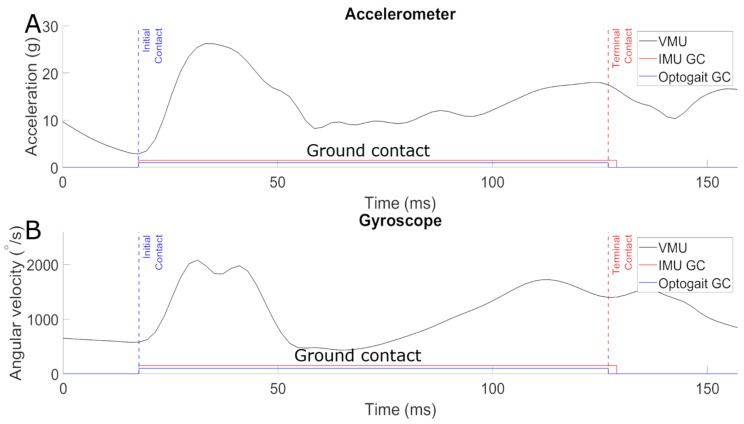
Vector magnitude unit (VMU) of x, y, and z acceleration (**A**) and angular velocity (**B**) throughout one single sprint step. The blue dashed line marks the initial contact event; the red dashed line the terminal contact. The solid red line indicates the resulting ground contact period for the inertial measurement unit (IMU). The photo-electric-measured (Optogait) ground contact time is represented by the solid blue line.

For algorithm development, repetitive patterns in the IMU signals were analyzed and used to extract GCT. Six randomly selected runs served as training data. After the development, no further adaptations were made to the detection, which was applied to the rest of the runs. This procedure helps ensure that the algorithm does not over-fit the data. Figure 2 exemplary illustrates the algorithmic determination of those time points for one single step of a sprint. The IC of the foot causes an abrupt change of the acceleration induced by the touchdown. In this study, IC was defined as the moment of the local minimum in acceleration when the heel impacts the ground (Figure 2A). Using this definition of IC, the location of TC time points was determined with the help of the ground truth data in the training set. The foot’s movement at the end of the contact phase causes two peaks in the combined angular velocity. TC was defined by the local minimum between these two bursts (Figure 2B). The graphs show synchronous signals of the accelerometer and gyroscope of the IMU for one single step, together with the Optogait signal for ground contact.

### 2.4. Statistical Analysis

Results regarding the whole sample include all steps which at least two athletes performed. This discrepancy comes from the different step lengths of the tested athletes. This leads to a maximum of 50 steps for any 100-m sprint.

All Graphs were created with Microsoft Excel (2016, Microsoft Corporation, Redmond, WA, USA). Percentage differences are calculated as the percentage deviation of the photo-electric measured value. To show error distributions, a well-known procedure is the visualization of data in a Bland-Altman-Plot. Step-wise deviations are indicated by means of root mean square error (RMSE) between the calculated GCT and the GCT of the ground truth.

## 3. Results

Section 3.1 addresses results on the validity of the GCT detection. Section 3.2 illustrates the distribution of the measured GCT. Moreover, based on IMU data, exemplary evaluations of individual runs regarding reliability and gender comparison are visualized. Results are shown as percentage values, averages, standard deviations, and Bland-Altman plots.

### 3.1. Results on Validity

The algorithm correctly detected 863 of 889 ground contact events, corresponding to a false detection rate of 2.92%; 6.47% of the first five steps and 13.33% of the last five steps of the respective sprint were incorrectly detected. The remaining sprint steps were incorrectly detected in 0.56% of the cases. The IMUs detected a mean GCT of 119.95 ± 22.51 ms, and Optogait detected 117.13 ± 24.03 ms for all simultaneously measured steps. The step-wise average relative time difference between IMU- and Optogait-GCT was 3.55 ± 6.16 ms, which translates to a 3.03% average deviation of GCT. A mean absolute time difference of 5.46 ± 4.55 ms (4.66% deviation) was measured. The deviation of each step results in a total root mean square error of 7.97 ms.

**Figure 3:**
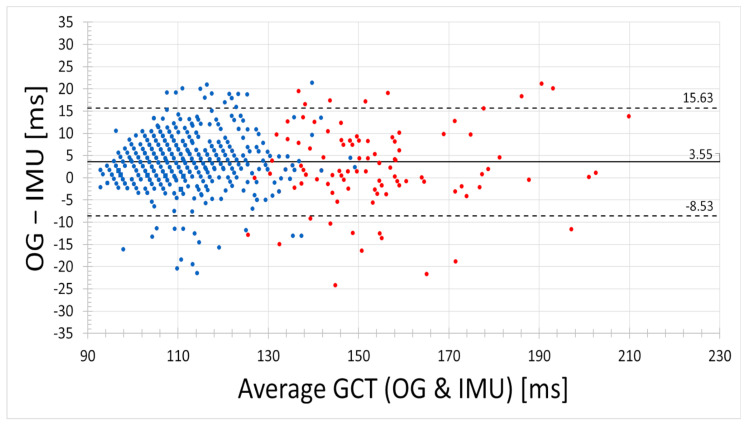
Bland-Altman-Plot of IMU- and Optogait (OG) measured ground contact time (GCT). Dashed lines show Limits of Agreement (2∗SD): −8.53 ms and 15.63 ms, the dotted line the mean: 3.55 ms. Red data points represent steps 1–5 at the beginning of the sprint. Blue-colored dots indicate all other steps (i.e., step 6–50).

Measurement errors for the detected GCT are illustrated in a Bland-Altman plot (Figure 3). All steps with both IMU and ground truth data are shown independently of the respective trial. The first five steps are marked with red dots, steps 6–50 are shown in blue color. The solid black line represents the mean bias of all detected steps: 3.55 ms. Limits of Agreement (2∗SD) were obtained at −8.53 ms and 15.63 ms and are represented by the black dashed lines.

**Figure 4:**
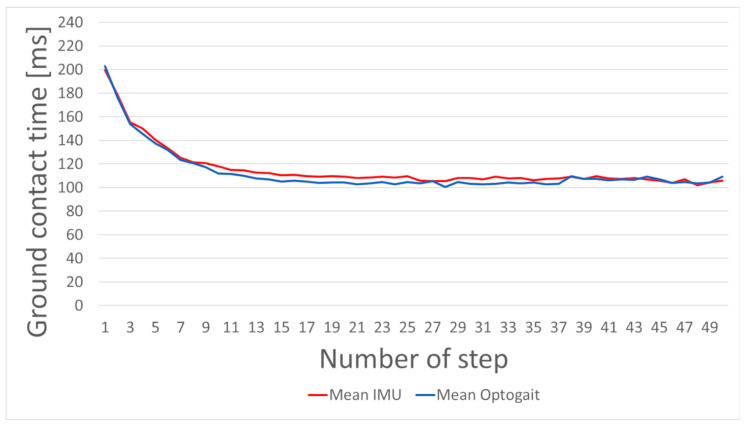
Average GCT of Optogait (blue) and IMU (red) measurements. Only these steps are included, which at least two different athletes performed. All measured data points are summarized to one value for the respective step in the sprint.

Figure 4 shows the average step-wise measured GCT with Optogait (blue) and IMUs (red).

Table 1 shows the distribution of GCT throughout step ranges of five and ten steps. The measured mean and absolute percentage deviation to the reference system in the respective step range is displayed. Steps 6–46 show a constant deviation in the range from 3% to 6%. The first five steps indicate a lower relative difference (1.17%). The absolute values are in the range above. For the last five steps, a lower relative (0.22%), as well as absolute (2.13%) deviation is found.

### 3.2. Results on GCT

The following result section illustrates the IMU-detected GCT of exemplary single runs. The first graph visualizes the reliability of the measured GCT by showing two runs of the same athlete. The following graph emphasizes the application of this method, comparing GCT from single runs of athletes from different genders.

**Figure 5:**
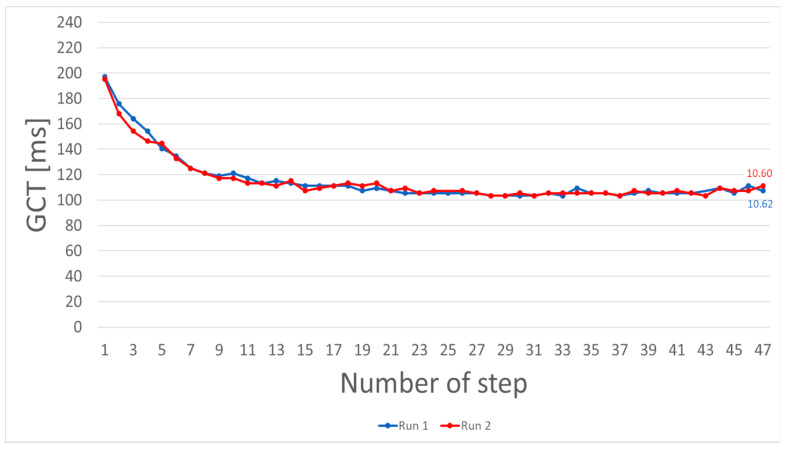
IMU-measured GCT of two 100 m sprints of the same athlete. Run 1 (blue) was conducted approximately 30 min before Run 2 (red). The graph illustrates reliable intra-subject results.

Figure 5 shows two separate sprints of the same athlete. Chronologically, blue represents the first and red the second sprint. The difference between the GCT of both runs is on average 0.48 ms per step For steps 1–5, an average decrease of 27.36% in both runs can be seen.

**Figure 6:**
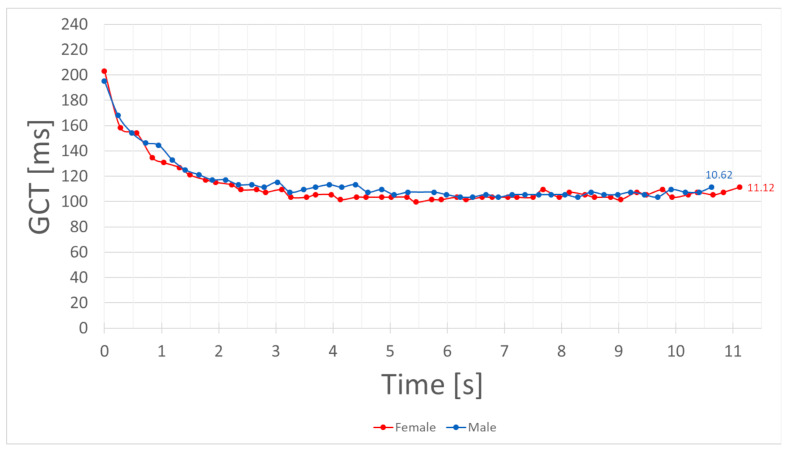
IMU-measured GCT of a female (red) and a male (blue) sprinter over 100 m. The marked dots on each line represent ground contacts. The connecting line between the dots is added for better visual separation. The time of the last contact represents the total period between the first and the last step of the respective sprint.

An exemplary comparison of GCTs of a female and a male sprinter is given in Figure 6. These two individual runs were chosen to illustrate the possibilities of this method. Each ground contact is represented by a dot on the respective line. The time between the last and the first step of this 100-m dash was 10.66 s for the male and 11.12 s for the female sprinter. The number of steps altered with 50 steps for the female athlete and 47 for the male athlete. No gender dependent differences occur in the top speed phase of the run.

## 4. Discussion

The current study was conducted to explore and prove the benefits of sensor-based running parameters in top-level sports.

### 4.1. Discussion of Methods

The detection of gait events from sensor data progressed in recent years. Various sensor outputs can be used to extract time points of interest. In addition, the procedure during data processing also plays a decisive role in the development. This study does not claim to extract the most precise or correct signals or features to estimate IC and TC. In other proposed methods, specific components of the accelerometer and gyroscope are tested for detection and applied to running signals [13,22]. Schmidt and colleagues used minima in an acceleration component of one direction to detect IC and TC. Falbriard et al. identified several different time points for IC and TC as possible ground contact indicators. They found a combination of acceleration and angular velocity as promising by comparing these time points to the detection of a force plate as the criterion reference system.

Especially for validation purposes, the study design should focus on an appropriate criterion measurement system. The criterion measurement system in the current study, Optogait, is a commonly accepted method for ground truth data acquisition [7,13]. The recent study’s setup—including the alternation of the 50-m corridor—was also used in other studies [7]. Additionally, the time-synchronization of all systems could improve the insights during the algorithm development. Although the burst in the accelerometer at IC was used to synchronize the events a posteriori by minimizing the least square errors of the first IC, it is possible to circumvent this error source with a technical synchronization. Hence, this time-synchronization would allow for a more accurate assessment of the IC and TC detection.

The results for GCT in Figure 6 are exemplary and must be further compared to inter- and intra-subject results. Additionally, more incorrectly detected steps occurred during the first five (6.47%) and last five (13.33%) steps. Therefore, improvements to the used algorithm should be achieved, especially since the methodology of pattern detection from IMU signals has been a fast developing topic in recent years; for example, the early steps of a sprint (see Figure 3) show greater discrepancies.

A relatively small sample was chosen for this study to represent high-level sprinters from different gender, age, weight, height, and other variations. However, it cannot be assumed to have covered all discrepancies within this population. Therefore, no conclusions about general differences can be drawn within this small sample size. For further quantification of elite sprint performances, a broader data basis should be established while considering that the evaluation of individual athletes should also be supported. With the increasing usage of IMU sensors in recent commercial products, future studies can also account for combining further sensory data to a more holistic acquisition of running parameters of professional athletes.

### 4.2. Discussion of Results

The basic aim of a run analysis with IMUs should include the correct detection of sprint steps. The results on correct step detection can hint towards the algorithm validity. This study showed a detection rate of 97.1%. All false detected steps were missed real steps, corresponding to false positive detections. 80.8% of these detection errors occurred within in the first and last five steps of the respective sprint. This could be explained by the different coordination patterns at the beginning and end of the sprint which lead to unstable waveforms and ultimately false positives. To the best knowledge of the authors, the detection rate is only explicitly stated in one other study. Schmidt et al. reported the correct detection of 95.7% [13]. Thus, the proposed method showed results that are in line or even better.

Figure 3 and Figure 4 show the distribution of measurement errors for each step of the sprint. The mean overestimation of 3.55 ms of IMU-based GCT detection compared to Optogait hints towards a systematic bias (Figure 3). As Falbriard et al. stated, correcting the GCT values based on previous results may help to achieve more precise results [22]. However, Figure 4 illustrates low deviations at the beginning and end of the sprints. This can also be seen in Table 1. The lower deviations of step range 1–5 and 45–50 indicate that an adaptive GCT recognition could be helpful. The first five steps occur especially important, as big relative changes can be observed in this time span (Figure 5. These indications need to be considered in a potential correction procedure.

The limits of agreement in the Bland-Altmann analyses of −9.28 ms and 16.14 ms (see Figure 3) appear to be relatively high compared to another study with a similar reference system, which stated lower bounds [13]. Besides a different detection of ground contact events, a possible reason could be the shorter contact time of elite sprinters who participated in this study. Also, a different running style of the individual participant could make a crucial impact because of the small sample size.

In Figure 5 and Figure 6, individual runs are shown for application purposes. The temporal resolution of GCT enables the visualization of minor differences during a single run. The illustration of a female and a male sprinter in Figure 6 does not support a general gender comparison. However, as the tested sample performs on a high level and therefore experience individual improvements in running economy, the use case can show possibilities of individual analysis with IMU data. This representation of results does not contribute to the validity measurement or quantification of the tested population. In the individual analysis and comparison of these single runs, we can see indicators for running asymmetries (e.g., Figure 5—steps 43–47). As these asymmetries lie within the detected measurement error, the pure indication of IMU signals can not solely be referenced. This analysis can more easily be transferred to competition, as no counterparty or athletes are distracted by wearing such light and small sensors.

## 5. Conclusions

The step detection rate with the IMU data showed high reliability, whereas the deviation of the measured GCT depends on the section of the run. The early and late stages of the sprints tended to have lower deviations between IMU and Optogait measured GCT. These findings point out additional technical difficulties, such as problems of the algorithm. In total, the results encourage the field use of IMUs as a potential method for step detection and measurement of GCT in high-level sprints. This analysis can help to enhance our knowledge about performance on the highest levels. The findings of this study encourage the implementation of IMU-based measurements in high-level sprint competitions.

## Figures and Tables

**Table 1:** Mean IMU measured ground contact time (GCT), and its relative and absolute percentage deviation to the reference system for various step ranges of all 100-m sprints. The first and last intervals are summarized into five steps. All other intervals combine ten steps. The last five steps showed the lowest percentage difference.

Step	GCT ± SD	% Diff ± SD	Absolute % Diff ± SD
ine 1–5	163.45	1.17%	4.33%
	24.73	1.77%	0.36%
ine 6–15	118.43	3.28%	4.61%
	9.45	1.52%	0.78%
ine 16–25	109.32	4.28%	4.98%
	6.40	0.52%	0.69%
ine 26–35	107.12	5.14%	5.72%
	9.12	2.18%	1.27%
ine 36–45	107.86	4.24%	5.86%
	9.01	2.27%	1.13%
ine 46–50	104.80	0.22%	2.13%
	6.71	1.26%	1.11%

## Data Availability

The data presented in this study are available in the article.

## Abbreviations

The following abbreviations are used in this manuscript:

GCT	Ground contact time
IMU	Inertial measurement unit
GPS	Global positioning system
LPS	Local positioning system
VMU	Vector magnitude unit
IC	Initial contact
TC	Terminal contact
RMSE	Root mean squar error
SD	Standard deviation
